# Reshaping the narrative: Tracing the historical trajectory of HIV/AIDS, gay men, and public health in Sweden

**DOI:** 10.1371/journal.pone.0298630

**Published:** 2024-02-22

**Authors:** Fredrik Nyman

**Affiliations:** Department of Psychology and Social Work, Mid Sweden University, Östersund, Jämtland, Sweden; University of Foggia: Universita degli Studi di Foggia, ITALY

## Abstract

The human immunodeficiency virus (HIV) emerged as an endemic health crisis in the United States during the early 1980s. Initially labelled a “gay disease” due to its prevalence among gay men, the spread of HIV led to widespread fear and moral panic, as there was limited medical knowledge on preventing its transmission. While HIV is often associated with Sub-Saharan Africa, this article focuses on Sweden, a pioneering nation that became the first to achieve the remarkable Joint UNAIDS/WHO 90-90-90 continuum in addressing the epidemic. However, despite this significant milestone, the punitive legislation and attitudes prevalent in Sweden have had a counterproductive effect on curbing the virus’s spread. Drawing upon a comprehensive triangulation of various data and sources on the evolution of public policy in Sweden, this article argues for the urgent need to reduce stigma surrounding HIV and AIDS. By undertaking further measures to combat stigmatisation, we not only have the potential to prevent the spread of HIV but also significantly enhance the quality of life for individuals living with the virus. An essential step in this journey is to eliminate the legally-enforced mandatory disclosure of one’s HIV status, which would mark a tremendous victory for all those affected. With limited evidence to support the effectiveness of criminalisation and penal laws, no longer being viewed as criminals for non-disclosure would be a monumental achievement, positively transforming the lives of people living with HIV and fostering a more inclusive and supportive society.

## Introduction

### From deadly disease to chronic condition

With mortality in the tens of millions, AIDS (acquired immunodeficiency syndrome) remains “one of the deadliest medical disasters in the past century” [1]. The syndrome came into recognition in 1981 as a result of a growing incidence of infections among young gay men, from which they failed to recover [2]. It was then discovered that the underlying cause was the human immunodeficiency virus, or HIV [3, 4]. As the virus spread, it grew to become both a biological and a political issue [5]. The primary means through which HIV is transmitted include unprotected sexual intercourse, the usage of tainted blood products or unclean needles, and transmission from a mother to her child [6, 7]. HIV and AIDS caused much fear and panic throughout the 1980s, as many people were dying with no immediate medical knowledge of how to prevent the syndrome or counteract the virus [8, 9]. Misinformation about the virus and how it spreads led to common beliefs about persons living with HIV—mostly behavioural, e.g., promiscuity and immorality, which have no scientific basis yet have been used to fuel stigma and discrimination [10–12].

HIV is a retrovirus that works by reverse transcriptase, whereby an enzyme translates the single-stranded viral RNA present into double-stranded DNA; or in more simple terms, it is a virus that can add genetic information to the DNA of a host cell [6, 7]. These characteristics and the rapid spread of the virus in the body make a cure very difficult. Yet, medical treatment has come a long way. In fact, antiretroviral therapies (ARTs) have proved so efficacious that after contracting HIV, progression to AIDS is increasingly rare in many parts of the world, especially in nations with access to antiretroviral therapy. [13–15]. With early treatment, what is named a “functional cure” is even theoretically possible [16, 17]. This refers to a biological state in which the body can manage to suppress the virus without HIV inhibitors. In other words: medically, there have been enormous positive developments, so that nowadays HIV is primarily seen as a chronic condition and not as a virus that causes AIDS and leads to death. Yet despite the medical advancements [4], the history with which HIV and AIDS were discovered (and the ways people were informed and responded to this information) has its own story. These stories are of vast sociocultural significance, especially as they provide insights into understanding how people assign meaning to pathogens—and with what consequences. Culture, fear, and prejudice play major parts in this process [18], and it is not without reason that academic publishers across health research have devoted many volumes and special issues to HIV/AIDS throughout the years [19, 20].

As many scholars previously have illustrated, people unremittingly tend to associate the AIDS pandemic with the area of Sub-Saharan Africa; countries like South Africa, Lesotho, Eswatini, Botswana, the Democratic Republic of Congo, Guinea Bissau, Gabon, and Cameroon more specifically, where HIV and AIDS are especially prevalent [5, 21, 22]. Back in 2018, an estimated 61 percent of new HIV infections occurred in this region, and as of 2020, more than two thirds of those living with HIV in the world reside in Africa [23]. South Africa explicitly has the largest population of people with HIV of any country in the world; at 7.92 million, which translates to 14 percent of the overall population [23]. Nguyen wrote that the contemporary “politics” of AIDS are not those of colonial times, “but they nevertheless demonstrate parallels in the way that foreign powers continue to intervene in Africa” [5]. No longer to ‘kill and conquer’—but instead to ensure that certain populations may live. As a humanitarian emergency, “AIDS now defines exception in epidemiological terms” [5]. The AIDS pandemic in Africa has not remained static, however; rather, it “has evolved and transformed over time” [24]. Iliffe’s [25] seminal and comprehensive history of AIDS in Africa provides not only examples of this, but the volume is also exemplary of the recent ‘historical turn’ in scholarly writing on HIV and AIDS [4, 24]. That said, it is not the task of this article to reproduce Iliffe’s definitive account. This article is not about HIV and AIDS in Africa and the author abstains from detailing it much further beyond this point. Instead, the present study focuses on a Western European country—namely Sweden where, in epidemiological terms [26–28], HIV/AIDS has never been a problem to the same extent it has in Sub-Saharan Africa.

### HIV in Sweden: A rationale for research

Annually, Sweden reports around 400 to 500 new HIV cases, predominantly encompassing instances where the infection was contracted prior to the individual’s migration to the country [29, 30]. Nevertheless, Sweden holds the distinction of being the inaugural country to fulfil the Joint UNAIDS/WHO 90-90-90 continuum of HIV care objectives. By the conclusion of 2015, Sweden had achieved the following: 90 percent of HIV cases were diagnosed, 99.8 percent of individuals were connected to care, and 95 percent of those who had undergone a minimum of six months of antiretroviral treatment maintained a viral load below 50 copies/ml [28]. As a comparison, it is estimated that around 8,000 people live with HIV in Sweden [31]—which corresponds to approximately 69 cases per 100,000 inhabitants, or 0.07 per cent of the population—whereas UNAIDS [23] have estimated that over seven million people live with HIV in South Africa (the epicentre of the HIV/AIDS pandemic). Moreover, in this geographical region, 63 percent of new infections are women—with young women (aged 15 to 24 years) twice as likely as men of the same age to be living with HIV [23]. However, information about HIV/AIDS and its occurrence in Sweden has led to stigma and social stratification, especially when it comes to the public image and civil rights of gay men (and other Men who have Sex with Men) [7, 9].

Historically speaking, and even now almost 40 years after the initial description of HIV, gay men are vastly overrepresented in the Western world when it comes to prevalence of HIV. While gay men in modern times are considered people at increased risk of acquiring HIV by the World Health Organization [32] with targeted and comprehensive services to follow, for many years this was not the case and it took a long time for gay men to acquire this position [8, 9, 33, 34]. This is especially true for Sweden [35, 36], where gay men failed to become one of the most prioritised (at-risk) prevention groups for many years; not until 2005, when the first National HIV/AIDS Strategy was drafted, was this truly enforced within legislation in Sweden [37]. First implemented in 2006, the National Strategy was then updated years later in 2017. The current strategy centres on three intentions: 1) evidence-based health promotion that targets both relevant ‘at-risk’ groups, as well as the general population; 2) early detection and treatment of HIV infections; 3) reducing the pervasive stigma associated with the virus is crucial, allowing individuals with HIV to openly share their status without fearing discrimination or mistreatment [37]. As of 2021, a motion to further develop the national strategy is currently being processed by the Swedish Parliament [38], which outlines a plan that seeks to achieve a 95-95-95 continuum of HIV care targets [39].

Taking this to heart, the aim of the study from which this article derives was to provide insights into how gay men have become known and depicted within bureaucracy and public policy in Sweden—as well as its related “artefacts” [40, 41]—with the discovery and outbreak of HIV. In many high-income countries, HIV is more prevalent among Men who have Sex with Men (MSM) than among the general population [42–44]. Through triangulation of cross-checking data from multiple sources [45], this article specifically looks at how gay men historically became associated with HIV in Sweden, and what this has meant for them as a sexual minority. Not only in terms of their actual bodies, but also their agency and autonomy to use and access social space and civil liberties such as privacy and solidarity. To put it in Watney’s [46] terms: this article revisits the notion of how *people*—rather than viruses—are the true victims (casualties) of HIV prevention strategies. Following this historical reconsideration, the article takes a look at more recent events and scientific outputs on HIV and stigma reduction, where the author comes to argue that a further step in stopping the spread of HIV could be taken through reducing stigma; especially as stigma can discourage people from both getting tested and getting timely treatment.

This article engages with two vital questions: 1) How has the historical association of HIV with gay men (and other MSM) in Sweden impacted their representation in bureaucracy, public policy, and social space, and what are the implications for their agency, autonomy, and access to civil liberties? 2) How can stigma reduction interventions play their part in stopping the spread of HIV?

## Study design and methods

This article stems from several sources, research data and material, and uses triangulation as a means of enhancing reliability and validity by cross-checking or cross-referencing the various data sets [45]. Triangulation helps to strengthen the validity and reliability of the findings by reducing biases and confirming or cross-verifying results through multiple perspectives and data sources. In this case, the article combines data from interviews, extensive archival research, and a rapid narrative review.

### Interviews

Firstly, the article partially draws from a mixed-methods case study conducted in Stockholm (Sweden) between 2014 and 2015, which sought to describe with an accuracy and sensitivity the actual public health policy-making process (focusing on HIV and AIDS legislation, specifically) from the perspectives of policy-makers and policy professionals, in their capacity as such [47, 48]. The author met with professionals from seven various government agencies and non-profit organisations [Table 1], and conducted a total of eight interviews (all in Swedish, with inserted quotes translated into English by the author) with self-selected members of staff. These persons were not interviewed in their capacity as private citizens but as spokespersons for their respective agency or organisation. The interviews centred on historical events apropos the process of public policy—not the interviewees’ personal actions, experiences, narratives, or opinions. Sensitive personal data (under GDPR) was neither asked for nor divulged or documented in any way. Nevertheless, while personal opinions were never divulged, all responses and quotes have still been anonymised as to maintain and protect personal integrity. In this article, the interview material features for contextual use to further inform the study. While the absence of personal narratives from persons living with HIV themselves perhaps seems odd, this was intentional, as the research aim remained clear from the start; i.e., to document the policy-making process from the perspectives of policy-makers and policy professionals (and thus, not citizens’ experiences of or responses to policy). The participants of this study did not give written consent for their interview data to be shared publicly, so due to the sensitive nature of the research, supporting data is not available. In ensuring the long-term accessibility and usability of the supporting data, the author adheres to best practices and standards for data preservation; such as those recommended by trusted organisations like DataCite and the Data Preservation Alliance for the Social Sciences (Data-PASS).

**Table 1:** List of the organisations interviewed.

NAME OF ORGANISATION	CORPORATE FORM	DESCRIPTION/MISSION
1. *Hiv-Sverige* (HIV Sweden)	NGO	A patient association that supports and brings together people who want to work to improve the living conditions of persons living with HIV.
2. National Federation of Noah’s Ark Associations (*Riksförbundet Noaks Ark*)	NGO	An HIV/AIDS service organisation in Sweden that works to limit the spread of HIV (and its impact) on micro, mezzo, and macro levels.
3. *Posithiva Gruppen* (The Positive Group)	NGO	Initially a patient group and social venue point for Men who have Sex with Men (MSM) living with HIV in Sweden, but now welcomes members of all demographics.
4. Public Health Agency of Sweden (*Folkhälsomyndigheten*)	Government agency	A Swedish government agency with national responsibility for public health. It falls under the Ministry of Health and Social Affairs and works to promote public health and to prevent illness and injuries through education.
5. Swedish Association for Sexuality Education (*RFSU*)	NGO	An organisation that works with public opinion formation on sexual and reproductive health and rights, as well as information and education about sexuality and relationships.
6. Swedish Federation for Lesbian, Gay, Bisexual, Transgender, Queer, and Intersex Rights (*RFSL*)	NGO	The largest organisation working for LGBTQIA+ rights in Sweden. Founded in 1950, it stands as one of the oldest LGBTQIA+ rights organisations in the world.
7. Swedish National Board of Health and Welfare (*Socialstyrelsen*)	Government agency	A Swedish government agency and the central national authority for social services and health services. The Board establishes norms by issuing provisions and general advice. It evaluates legislation and activities conducted by municipalities, county councils and local authorities.

### Archival research

Secondly, for this article the author has also analysed relevant history apropos HIV and AIDS in Sweden through extensive document and archival research at the National Library of Sweden, in Stockholm (where non-digital data supporting this study are curated), as to provide an in-depth understanding and analysis of the subject matter [40, 49]. The policy documents analysed were grounded in three Swedish Codes of Statutes: 1) the *Act on Banning Sauna Clubs* [50]; 2) the *Communicable Diseases Act* [51–53]; and 3) the *Act on Blood Safety and Quality Regulations* [54–56]. This process did not just involve studying the said Codes of Statutes themselves but also the overall policy-making process; the bureaucracy, legal documents, and ‘coloured’ papers [57] that envelops them [40, 41] as well as the public debates that led to these Codes being enforced, including public media and newsprint articles published between 1981 and 2013 (primarily focusing on the largest newspapers in Sweden; *Aftonbladet*, *Expressen*, and *Dagens Nyheter*). This was done to gain insights into the process and transition of legislation, as well as to unravel what type of knowledge of HIV itself—and the persons living with it—was used to allow for the bureaucracy inflicted upon gay men to take its shape. Whereas not always explicitly shown through citations or the equivalent, the documents and archival records employed in this study underpin the analysis.

### Rapid narrative review

Thirdly, a rapid literature review [58] was performed, seeking to provide the best complimentary evidence in response to the topic at hand (alongside the interviews and archival research). An important aspect that differentiates rapid systematic reviews from traditional systematic reviews “is the more marked need to support production across the review’s lifecycle, from early question generation and method planning to development of the manuscript” [58]. The speed at which a systematic review is conducted does not necessarily indicate its quality. It is possible to complete the same amount of work with the same level of quality in a shorter or longer timeframe. However, it is important to note that rushing through a review may lead to compromises in the overall process. This can include overlooking important evidence and making errors in the assessment or synthesis of the available data. Therefore, rapid reviews should still adhere to the core principles of systematic reviews to ensure unbiased inclusion, assessment, and synthesis of studies.

Systematic reviews are considered the ‘gold standard’ for the location, appraisal and synthesis of research evidence in relation to a given question [59], especially as their explicit, transparent and rigorous methodology aims to synthesise findings from all relevant research that meets their inclusion criteria [60]. Rather than ‘systematic’, however, in the strict sense of the word, the literature review performed for this article draws inspiration from Mason and Sultzman [61] in making it a narrative review (yet taking a reasonable systematic approach to searching the literature and reporting it). Narrative reviews, as such, typically begin with a defined question to be addressed; however, they often involve a more general discussion of a subject without a specific hypothesis, adopting a topical approach instead. They cover a broader range of topics compared to systematic reviews, offering greater insights into questions where not all contextual factors are known in advance or where the historical context is considered relevant [62].

To obtain a comprehensive understanding of the literature, an extensive search was conducted using specific search terms in MEDLINE, PubMed Central, Scopus, and Web of Science. The search string employed was designed to encompass various aspects of the topic under investigation. The primary search string used was [[AIDS or HIV] AND [gay or homo* or MSM] AND [stigma or social* or emotion* or psychologic*]] AND [Sweden[Title] OR Swedish[Title]]. To ensure a more inclusive search, a slightly broader search key was also utilised: [MSM [AIDS or HIV] Sweden]. This expanded search string aimed to capture a wider range of relevant articles and information. In addition to the electronic searches, a thorough examination of the references cited in the identified articles was conducted. This approach, known as backward and forward citation searching, allows for the discovery of additional relevant literature [63, 64]. Supplementary sources, including semi-scientific and official documents, were also sought to enhance the breadth of the research. Google Scholar, LIBRIS (the Swedish national union catalogue maintained by the National Library of Sweden), and the Swedish digital repository *Digitala Vetenskapliga Arkivet* (DiVA) were consulted. The latter platform provides access to publications from Swedish universities, university colleges, public authorities, research institutes, and museums, thereby facilitating the retrieval of openly available materials. By employing these comprehensive search strategies and utilising multiple resources, an extensive range of literature was gathered to establish a solid foundation for the study.

**Fig 1:**
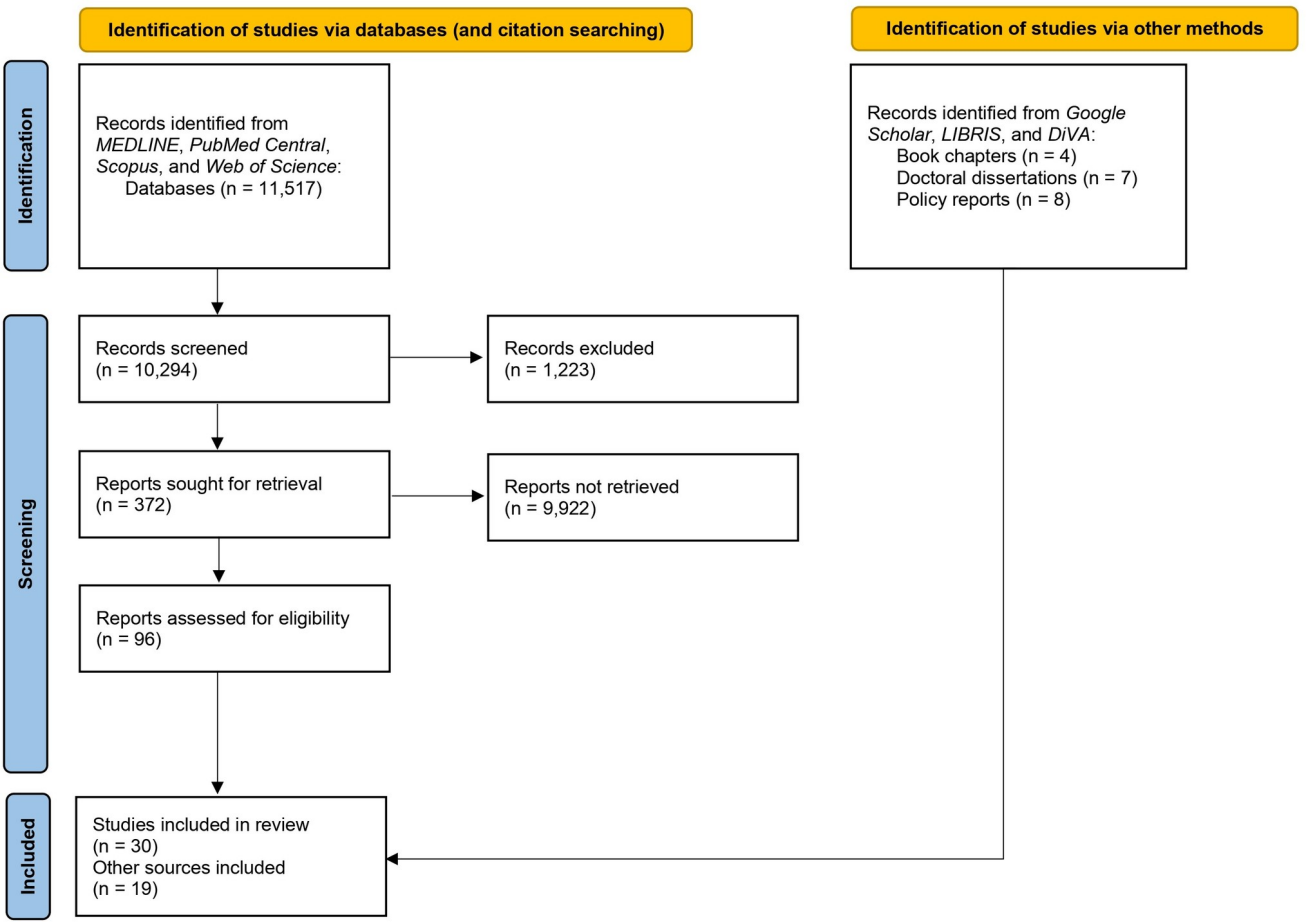
Flow chart of literature search results.

The search yielded a total of 11,517 individual records, out of which 372 were identified as potentially relevant [Fig 1]. From these, a total of 96 studies were found to meet the inclusion criteria. Due to the large number of included studies and the extensive volume of data, it became necessary to systematically categorise the results. This review focuses on studies that have examined context-specific historical events and culturally influenced processes related to HIV and AIDS, as well as the heightened risks faced by gay men (or MSM, more broadly) in Sweden over the years. Consequently, the author selected 30 studies for inclusion in this study. Additionally, 19 other sources were utilised (including semi-scientific materials) such as book chapters, dissertations, and policy reports (thus, in total 49 records; Table 2). These additional sources were incorporated because the scientific literature often lacked descriptions of pertinent historical events, necessitating the use of supplementary resources to provide a comprehensive contextual understanding. Data sharing is not applicable to this article as no new data were created or analysed in this study.

**Table 2:** Presentation of the selected records.

AUTHOR/YEAR	DOCUMENT TYPE
1. Swedish National Commission on AIDS (1986)	Policy report
2. Finer (1988)	Policy report
3. Henriksson (1988)	Policy report
4. Henriksson (1990)	Policy report
5. Månsson (1990)	Journal article
6. Henriksson & Ytterberg (1992)	Book chapter
7. Malmquist & Ramgren (1992)	Policy report
8. Henriksson (1995)	Doctoral dissertation
9. Eriksson et al. (2000)	Journal article
10. Herlitz & Steel (2000)	Journal article
11. Svéd (2000)	Book chapter
12. Albaek (2001)	Book chapter
13. Ljung (2001)	Doctoral dissertation
14. Walden Laing (2001)	Doctoral dissertation
15. Andersson (2003)	Book chapter
16. Tikkanen & Ross (2003)	Journal article
17. Kulick (2005)	Journal article
18. Herlitz (2007)	Policy report
19. Jallow et al. (2007)	Journal article
20. Bredström (2008)	Doctoral dissertation
21. Christianson et al. (2008)	Journal article
22. Berner (2011)	Journal article
23. RFSL (2011)	Policy report
24. RFSU et al. (2011)	Policy report
25. Thorsén (2013)	Doctoral dissertation
26. Albert et al. (2014)	Journal article
27. Ross et al. (2014)	Journal article
28. Persson et al. (2015)	Journal article
29. Schönnesson et al. (2015)	Journal article
30. Mehdiyar et al. (2016)	Journal article
31. Persson et al. (2016)	Journal article
32. Petersson et al. (2016)	Journal article
33. Gisslén et al. (2017)	Journal article
34. Carlsson-Lalloo et al. (2018)	Journal article
35. Persson et al. (2018)	Journal article
36. Reinius (2018)	Doctoral dissertation
37. Schönnesson et al. (2018)	Journal article
38. Andersson (2019)	Doctoral dissertation
39. Dennermalm et al. (2019)	Journal article
40. Herder & Agardh (2019)	Journal article
41. Zeluf-Andersson et al. (2019)	Journal article
42. Andersson et al. (2020)	Journal article
43. Herder et al. (2020)	Journal article
44. Mehdiyar et al. (2020)	Journal article
45. Carlsson-Lalloo et al. (2021)	Journal article
46. Dennermalm et al. (2021)	Journal article
47. Ehrenkranz et al. (2021)	Journal article
48. Reinius et al. (2021)	Journal article
49. Nyman & Jellesma (2022)	Journal article

### Analysis

Through the use of a thematic analysis [65] of the interview data, archival research, and retrieved literature records (in triangulation), three overarching themes were identified. These have been named as follows: 1) *From a limited problem to a disease dangerous to society*, 2) *Gay men as ‘outliers’*, and 3) *A counterproductive approach*. The first theme delves into the comprehensive evolution of HIV in Sweden, tracing its progression from being perceived as a limited issue primarily affecting gay men) and MSM, more broadly) to a disease that posed a significant threat to society as a whole, thereby warranting public concern. Moving on, the second theme sheds light on the complex portrayal of individuals with HIV and AIDS in Sweden, showcasing a dual perspective where they are recognised as victims of a deadly illness, yet simultaneously depicted as potential dangers to those unaffected by the virus. Lastly, the third theme explores the counterproductive approach specific to Sweden, wherein individuals living with HIV have not only faced blame for their condition but have also been burdened with sole responsibility for preventing its further transmission. These three themes are interconnected, highlighting the importance of reducing stigma to effectively curb the spread of HIV.

## Results

### From a limited problem to a disease dangerous to society

The first case of HIV in Sweden was officially diagnosed in August 1982. Like the first cases found in North America the patient was a gay man, named Roar Klingenberg [34]. Klingenberg was hospitalised soon after disclosure and died two years later in September 1984 [34]. As history shows, upon the appearance of HIV in Sweden public opinion wavered between trust and mistrust in the medical community’s capacity to keep the (*potential*) epidemic under control [9, 34, 66]. Public media outlets were also divided—between those who expressed faith in medical science and those who showed distinct distrust towards it, much due to how the rendered projections of the actual quantity of HIV infections in Sweden, at the time, were characterised by significant uncertainty [7, 67–69]. Even though medical experts were still vastly unsure of what was causing the syndrome (AIDS), or how it was transmitted, clear signs of optimism were still expressed in some segments of Sweden. Various newsprints urged people not to give in to despair, stating that the medical expertise was doing everything they could to come up with a potential solution [9, 34, 67, 70]. By the year-end of 1983 and in early 1984, the Swedish National Board of Health and Welfare went out with the announcement that the risk of an AIDS epidemic breaking out in Sweden had now ‘officially’ passed. This announcement was vastly criticised and later had to be retracted, however [34, 70]. Yet it does show, as Ljung [67] argued, that the Swedish authorities did not expect AIDS to develop into the societal issue it in retrospect did. Thus, in summary, the early epoch in Sweden was distinguished by HIV and AIDS being seen as a relatively defined and limited problem [34, 68, 71–73] that would solve itself without any interference. This would explain the political decision of not ‘combating’ the potential epidemic with nothing but limited resources and inputs [74, 75]—which is not to be considered characteristic of Sweden, but rather, something archetypical of Western Europe altogether [44, 74, 76–78].

This consensus changed, however, in February 1985, when a young boy suffering from haemophilia contracted HIV from a unit of blood plasma given to him at a Swedish hospital. In response to this incidence, Swedish Federation for Lesbian, Gay, Bisexual and Transgender Rights (RFSL) urged gay and bisexual men not to donate blood, and as documented, Sweden was the only place where such approbation took place [8, 9, 68, 69, 73, 79]. The gay federations in the rest of Europe immediately rejected such ideas, as they saw it as nothing but yet another act of discrimination where biological phenomena were used for moralising purposes in segregating sexual minorities [9, 68, 72, 80, 81]. The Swedish National Board of Health and Welfare hesitated for a long time before they decided on how to act, and even expressed lack of vital medical knowledge needed to make such a call. As seen reflected in the legislation and policy documents from back then [66, 69, 71, 82], which the author had access to through the archives at the National Library of Sweden (and also managed to elaborate on in interviews with various policy professionals), at first, the National Board was afraid of turning blood donors away. Thus, they chose to act rather passively by not defining any specific groups being at risk. However, the National Board later changed direction, where they later started the said culling of previous ‘at-risk groups’—stating that these individuals were no longer welcome as blood donors. This did not, however, refer to gay or bisexual men explicitly, but rather, sexual behaviours with multiple partners, which were a relatively small amount of people [9, 34]. Following this, the National Board later implemented a strategy that involved directives formulated as to restrict access even further, with the definition of ‘risk’ now including all gay and bisexual men who have had sex after 1979 [9].

This deed could, Ljung argued [67], be seen as a way of associating the risks of blood transfusion with the ‘at-risk’ groups believed to be causing the spread of the virus. HIV was now considered a disease that originated from gay and bisexual men [9, 34, 68, 71, 83], and thus a medical condition that could easily be transmitted to the rest of society if contact with these said groups was made. It did not take long before HIV/AIDS was deemed worthy of national attention due to the revelation that masses outside of the previous named ‘at-risk’ groups were now in danger of being affected, as well. With this, AIDS was no longer the enigmatic disease only befalling ‘the others’—but something that concerned society at large and demands of immediate action was soon raised across the nation. This was reinforced by the fact that the number of documented HIV infections in Sweden almost tripled—from 324 to 968 [70]—partially due to newfound ways of diagnosing whether someone was affected by HIV or not [9, 73]. AIDS increased as a newsprint topic around the same time, with papers headlining it all as the ‘new threat’ while also personating the groups vastly associated with the syndrome—i.e., gay and bisexual men; sex workers; People Who Inject Drugs (PWID); African migrants; and people who had contracted AIDS through blood products [7, 34, 67]. However, it should also be noted that a substantial hetero-sexualisation of AIDS had started to occur [9, 34, 72], where prevalence among heterosexual men and women had now increased. With AIDS now being of public concern, no one was deemed safe from infection.

### Gay men as ‘outliers’

Because of this, people living with HIV and AIDS came to be associated with a rather ambivalent image. On one hand, they were seen and acknowledged as unfortunate victims of a painful and deadly disease—on the other; they were construed as dangerous perpetrators, threatening those still unaffected by the virus [9, 67, 70, 71, 83]. Fear is also seen reflected in metaphors people have used for AIDS, such as ‘war’, a gay ‘plague’ or ‘cancer’, ‘hell’ or even ‘the wrath of God’ [70, 84, 85], which sometimes included a moral charge against those who had contracted HIV and were already stigmatised (such as gay men and PWID). In Sweden, drastic measures of all sorts were suggested; from implementing “sexual passports” (stating the health condition and HIV status of its carrier, which needed to be renewed regularly) to confining people to special ‘camps’ or tattooing them in the axilla, aiming for simple ways to identify persons suspected of living with HIV or AIDS [67].

Needless to say, none of these proposals were enacted by the Swedish Parliament. However, that said, a Swedish Act banning gay sauna clubs was voted through. As Bérubé [86] wrote, gay baths and bars are integral parts of gay political history. They contradicted the stigmas of gay people as “sinners, criminals, and diseased”—giving them a sense of pride in themselves and their sexuality [86]. In the United States, gay baths and bars became “the first stages of a movement of civil rights” [87] for gay people. Before there were any openly gay political leaders or organisations, “pioneers” (as called) spontaneously established gay bathhouses/saunas and gay/lesbian bars; risking arrest and jail sentences, even losing their families or their jobs, as well as putting themselves in potential danger, in order to transform public bars and baths into safety zones “where it was safe to be gay” [86]. The gay bathhouses therefore represent a major success in a political struggle to overcome social isolation and to be able to develop a sense of community and pride; a struggle that has been going on for centuries, and still must be won on many areas [86, 87]. It was during the late nineteenth and twentieth centuries that public baths and health resorts started to transform into the gay institutions they have come to represent today. Back in these days, all sexual acts between men were seen as ‘crimes against nature’; that is, they were considered and treated as public and illegal offences [88]. Thus, men having sex with other men had no legal right to privacy and were therefore forced to become sexual ‘outlaws’ [86]. In response to this, gay men became experts “at stealing moments of privacy” [87] and finding so-called ‘cracks’ in society, where they could meet without risk of getting caught, or judged. This is comparable to what Wright [89] called “refuse spaces”; unwittingly ignored sites of varying shapes and sizes, or peripheral areas on the margins of cities. In general, the proposition of banning gay bathhouses and sauna clubs was not just seen as an attack on the actual venues, but also on the gay community at large.

According to one interviewee—a delegate of *Posithiva Gruppen* (PG)—this certain Act that banned sauna clubs was the result of a demoralisation of the gay scene, initiated by the Swedish press. As the author was informed, it all began in October 1986 when a man named Peter Bratt, who was working at *Dagens Nyheter* (a major Swedish daily newspaper), started to publish a series of newsprint articles.

**PG**: Many remember it to this day. It was due to some articles published by Peter Bratt, a reporter at *Dagens Nyheter* [DN]. These articles basically led to the legislation of the Act on Banning of Sauna Clubs. Bratt visited Manhattan [an old cinema located at Hantverkargatan in Stockholm] and was allegedly shocked over what people were up to. It was rather upsetting what he wrote, even to this day. Well, DN acted quite disturbingly in overall during the 1980s, but Bratt especially. In fact, I remember the exact date; it was published on 10^th^ October 1986.

**Author**: And it was a big coverage?

**PG**: Oh yeah, it was huge. And it basically… it was after it was published that the Act on Banning of Sauna Clubs was set into motion. It was implemented only one year later.

**Author**: So, he was not involved, so to say, “officially” … yet it was based on and emerged out of something he wrote, and published?

**PG**: Yes, that is exactly how it was. No, Bratt was just a reporter working at DN. He was quite young back then. And AIDS was huge at the time—when it had just erupted in Sweden. He is quite an interesting person, though. Basically, the whole legislation is based on his… not interviews, but he was there, kind of doing “undercover work” at the gay place, you know.

In this way, gay men, who were most often affected by HIV, were portrayed in a demoralised way, which led Swedish authorities to focus on protecting society at large against those individuals that were seen (and portrayed) as spreading the virus. The changes within the Communicable Diseases Act reflected a similar shift in focus. This legislation existed before HIV and AIDS were discovered, but was modified in response, first in 1988 [51, 52]. The notorious result was an Act which 1) allowed for persons living with HIV to be put under indefinite isolation; 2) forced a statuary duty upon them, urging them to inform their sexual partners about their HIV status; and 3) obliged their physicians to without delay contact the health authorities every time a new case of HIV was discovered [71, 90, 91]. In sum, the outcome was that ‘at-risk groups’ were directly held responsible for their own infection. They were set apart from those persons seen as ‘innocently infected’; that is, those who contracted the disease through blood products, blood transfusions or from birth [7]. This also becomes clear in the following quote from the Swedish National Commission on AIDS:

There is a reason why HIV infection has spread among certain groups, above all homosexual men and injection drug abusers. It is due to a certain kind of behaviour—multiple partners and, for example, anal sex or drug injection with contaminated needles—that the infection has spread among these groups. And one must of course distinguish between those who have been infected by blood or blood products and those infected by risk behaviour. Although it is behaviour, not group identity in itself, that aggravates the risk of infection, we have elected to speak in terms of groups at risk. This concept has the advantage of leading to a classification of target groups [82].

The Act was soon widely known for its harsh nature and regulations, which were unique for Sweden at the time and not found elsewhere in the world. Not only did the Act violate the recommendations laid forward by the WHO but also those of the Council of Europe; all built on the free will and solidarity of both citizens and non-citizens [36, 68, 71, 72, 90].

As the attitude towards HIV and AIDS in Sweden changed during the 1990s, so did the need and wish for a more attentive Act showing significant concern for persons living with HIV and their life situation. Thus, the Government of Sweden issued (after severe pressure from the gay movement and various non-profit organisations) a re-valuation of the legislation [36, 70, 90]. The Act on Banning Sauna Clubs was officially abolished on 7^th^ April 2004 following the implementation of the currently actualised Communicable Diseases Act [91]. This was eagerly anticipated, much appreciated and welcomed by the various NGOs engaged in HIV prevention and sexual health across Sweden [35, 36]; largely due to its alleged counterproductive nature, as health authorities in Sweden purportedly understood that it would hold no practical significance in curbing the spread of infection. Instead, authorities aimed to demonstrate political resolve, mirroring how the law was expedited amid a debate marked by apprehension about the unfamiliar and revelations about recent discoveries regarding HIV and AIDS [70, 71]. In fact, right from the outset, NGOs like RFSL, RFSU, and *Venhälsan* (a sexual health clinic in Stockholm for Men who have Sex with Men) had asserted that imposing a ban on sauna clubs would yield counterproductive results. They contended that the activities prevalent within sauna clubs would simply relocate to other settings, complicating efforts to disseminate information about infection risks [70]. Despite articulating these concerns, their input was unfortunately disregarded by health authorities [64, 70, 71]. Even now, concrete statistics remain elusive regarding the actual number of individuals in Sweden who contracted HIV at a gay sauna club [71]. This perhaps underscores the moralistic undertones prevalent during a period characterised by substantial uncertainty [9, 34, 67].

That said, what is of analytical interest here, especially with regards to the Act on Banning Sauna Clubs, is the aspect of time—and the time it takes for bureaucratic implementation to actually commence. Hage wrote that there is politics around “what waiting entails” [92]. Waiting defines class and status relations in, as Hage phrased it, “the very obvious sense of ‘who waits for whom’” [92]. With regards to the Act on Banning Sauna Clubs, it only took four months for the proposition [93] to go from being just that, into becoming a vigorous Act within Swedish legislation. However, when it came to the discussion of a potential withdrawal of the Act, the process spanned over two decades. Still, within the most recent Communicable Diseases Act [53], there is still a ‘duty to inform’ scripted and persons living with HIV who do not submit to the duty-to-inform may be put under coercive isolation.

At the same time, an extraordinary case surfaced in 1997 where an African/American man residing in Helsinki (Finland), was suspected of having infected several Finnish women with HIV [67, 70]. Soon following, revenge became a recurring theme in Swedish media where persons living with HIV or AIDS were depicted as vastly vindictive individuals who knowingly transmitted the virus to other people [9, 34, 67, 71, 72]. However, while cases of persons who intentionally infected others with HIV made the news in several Western European countries [94, 95] it has never been examined whether these reports have influenced people’s attitudes about others with HIV. Moreover, in Sweden specifically, these cases have tended to revolve around heterosexual men [7, 67, 71, 96, 97] spreading the virus to women, while HIV is predominantly found in gay men and other MSM. As such, while intentional transmission of HIV has been deemed a criminal act without bias, at large such policies have mostly come to affect the MSM population—or even persons completely unaware of their HIV status [64]. This is why criminalisation for long has been seen as counterproductive by NGOs in Sweden, as it ultimately comes to affect the wrong persons [34–36, 67, 98–100].

Interestingly, the concept and social category of ‘Men who have Sex with Men’ (MSM) has been in use in HIV-related literature since at least the end of the 1980s, and the beginning of the 1990s, whereas the acronym of ‘MSM’ was coined and implemented around year 1994 [8, 101–103]. These terms “held the promise of reducing AIDS stigma” [102], which over the years had become increasingly attached to primarily gay men, but also lesbian women, and users of the terms “helped to promulgate these now-familiar acronyms” [102]. Yet scholars are troubled by how the acronyms are applied, since it seems, they have come to “displace rather than coincide” [102] with information about sexual identity. From available documentation, Boellstorff [104] stated that it seems clear that the category of MSM, like the ‘homosexual’ [105], did not originate from any household or bar, park or disco (et cetera), but instead through scientific and bureaucratic coinage [104]. It was created to signify behaviour without identify, such as in the phrase: *men who have sex with men but do not identify as gay*. The category did not emerge out of pride marches or mass media, but rather, in response to a need to analytically describe men (for purposes of HIV/AIDS surveillance and behaviour change) who engaged in intercourse with other men, but who did *not* identify *as* gay [104]. According to Boellstorff, the MSM acronym was meant to invoke behaviour in complete distinction from identity—as in an epidemiological imaginary, behaviour could stand alone. That is, a gay-identified man who lives in celibate is *not* at risk for sexual transmission of HIV, while a straight-identified man who has had sex with other men, in fact, *is*. He is offered no protection from infection whatsoever by the mere fact of his self-identification; “it is not who you are, it is what you do” that matters [104]. However, with the legislation in Sweden, a monogamous gay man is seen and defined as in higher risk of contracting HIV than a ‘straight’ man with multiple sexual partners. Even when this gay man is HIV-negative and in a relationship with another HIV-negative man, both would be considered ‘risky’ due to gay men being so vastly overrepresented when it comes to cases of HIV. Also, sex among homosexuals is not distinguished from another but always classified as ‘unsafe’; which is to say, with the current legislation, there is nothing called ‘safe sex between homosexual men’ [7, 9, 70–72].

As a result of these processes, gay men have become portrayed as scapegoats instead of being treated as a particularly “hard-hit group” [7]. Moreover, they are also held responsible for preventing the spread of HIV [7, 9, 34, 35, 67].

### A counterproductive approach

Recurring in Swedish history is the description of passing on the virus (HIV) as a criminal act. Persons living with HIV are not only blamed for having it, but also given the sole responsibility to prevent the spread. An example of opinion from this debate can be found in *Dagens Nyheter* (DN) from 2011, where Christer Winbäck (a trained nurse and a former Member of Parliament for the Swedish Liberal Party) commented on the present-day situation of persons living with HIV in relation to Swedish legislation, civic duties and responsibilities, as well as potential penalties:

HIV is undeniably a severe illness, and it is crucial to establish appropriate measures to address it. Consideration should be given to situations where individuals are unaware of their infection, as well as cases where transmission is intentional, warranting stricter penalties. Granting impunity without scrutiny would provide everyone with the option to claim ignorance. However, a careful evaluation must be conducted to determine the likelihood of the person’s knowledge regarding their HIV status [106].

Winbäck [106] points to several things with his statement. Firstly, he refuses to abolish HIV transmission as a criminal offence, and this with no regard to whether the person in question knows about their condition. That is, Winbäck sees HIV as such a severe predicament that it should be assigned great penalties, if set health regulations are not met or upheld. Also, in how he discarded of a potential introduction of impunity, Winbäck seems to believe that persons living with HIV would cease to assume responsibility for their conditions in case it would not be a criminal act of doing so. That is, according to Winbäck, Swedish legislation at large is in great need of a directive which states that persons living with HIV can be severely punished if they would not take their illness in full earnest. Since 1985, persons with HIV in Sweden have been obligated by law to disclose their medical status to potential sexual partners [i.e., ‘duty to inform’], with penalties for non-compliance [70, 90]. This legislation [51–53] allows for Swedish health authorities to request for mandatory detention and isolation of an individual if there are compelling reasons to believe that the person will not adhere to the rules of conduct, posing a tangible risk of infection to others [35–36]. By supporting a ‘duty to inform’ Winbäck is not only displacing the institutional responsibility befitting a state, but he also strives to maintain uneven and heteronormative structures which dissolve the aspects of equality when it comes to living with HIV or AIDS. Structures which hold some people liable for the misfortune of the ‘innocent’ ones, while also refusing to acknowledge the same groups as the victims or sufferers they truly are [35, 36, 71, 72, 91, 107].

Debates on whether it is time to re-valuate the Communicable Diseases Act occur several times a year and especially in adjacent to Stockholm Pride and the World AIDS Day. In fact, RFSL and Swedish Association for Sexuality Education (RFSU) have for a long time urged Swedish legislators to address the issues of the ‘duty to inform’, in hopes of breaking the stigma bound to HIV and thus improve prevention work [35, 36, 108, 109]. What these organisations hope to achieve, allegedly, is a society where more people dare to test and screen themselves for HIV, and thus, take further responsibility in practicing safer sex. Looking to the present-day situation, those who contract HIV in Sweden have, in most cases, gotten it from someone abroad and/or by someone who does not know they are a carrier [7, 72, 110, 111]. However, as Veronica Palm (a former Member of Parliament for the Swedish Social Democrats) has said, the current legislation risks ending up contra-productive, especially considering how it preserves a stigmatic image of persons living with HIV rather than working on facilitating and improving their position within Swedish society [91]. Also, as Case and colleagues demonstrated [112], studies show that prosecution of HIV transmission does, in fact, *not* reduce the spread of HIV, which has also been pointed out by RFSL, RFSU and other NGOs. As such, criminalisation acts yet as another proof of contra-productive legislation, in how it continues to focus on the wrong masses [35]. Moreover, as stated in the joint policy recommendation by RFSU, RFSL, and HIV-Sweden [36], the issues are not only found in the Communicable Diseases Act but also in Swedish Criminal Code.

The detrimental impact of the current situation extends beyond individuals living with HIV. We hold a strong conviction that specific clauses within the Communicable Diseases Act, such as the “duty to inform”, when coupled with provisions from the Swedish Criminal Code, detrimentally impact HIV prevention efforts across Sweden and additionally contribute to the stigmatisation of individuals living with HIV. We recognise that the Communicable Diseases Act was established with good intentions, aiming to prevent the spread of HIV and uphold equal rights. However, there are sections within it that do not align well with the National Prevention Strategy or create a sense of safety and comfort for individuals living with HIV to openly disclose their status. The time has come for a thorough review and evaluation of the Communicable Diseases Act. This audit should also encompass an examination of the Swedish Criminal Code, which governs the transmission of HIV between individuals [36].

Transmitting HIV to another person, or exposing a person to HIV, is not an offense in the Swedish Criminal Code in and of itself. Instead, people may be prosecuted and sentenced for various violent crimes (i.e., assault). The act itself is not sufficient, however; prosecutors must illustrate that the person acted with intent or with negligence [36]. With respect to the Swedish National HIV/AIDS Strategy, it states that stigma and discrimination related to HIV have to be minimised, and that persons with HIV can talk about their status without fear of being treated differently [37]. The counter-productivity of the ‘duty to inform’ has previously been further enlightened by RFSU, in how they said that it risks ‘lulling’ people in a false sense of security [34, 67, 68, 71–73, 91, 98, 110]. That is, if persons living with HIV are obliged by law to tell their sexual partners about their HIV status, how are people supposed to react when their partners do not say anything? They might assume that the person in question is HIV-negative, even though they can as easily be unaware of their HIV status. Further examples of this were given in an interview with a delegate of RFSU:

With the advancement of our understanding regarding infectivity, individuals living with HIV now have the option to request the removal of [the duty to inform] from their general practitioner. The Communicable Diseases Act encompasses various provisions, and the so-called ‘duty’ is just one of them. However, it is now possible to have this obligation waived on an individual basis, provided that the person is undergoing effective treatment and maintains undetectable levels of the virus. Nevertheless, at RFSU, we have always held a critical stance towards this supposed ‘duty to inform,’ as we believe it can create a false sense of security. We now know that those who are aware of their HIV status are not the ones posing a significant risk of transmission; rather, it is those who are unaware. Hence, we argue that the so-called ‘duty to inform’ is essentially meaningless. We strongly believe that removing this obligation would be a prudent course of action, echoing the wise words of someone from long ago: “Responsibility is not divided 50/50, but rather 100/100.” We do not deny the immense responsibility borne by individuals living with HIV. We recognise that many of them take it incredibly seriously. However, at RFSU, we refuse to accept that a specific provision within the Communicable Diseases Act would effectively prevent the spread of HIV.

Medical science has made immense progress when it comes to preventing HIV from developing into AIDS, turning this deadly disease to a chronic condition [14]. Back in 2008, the Swiss National Commission on AIDS caused quite a stir when they published some recent research findings. The results indicated that persons living with HIV who were under effective anti-retroviral treatment (ART) showed less risk of infection and should by that fact be considered sexually non-infectious [113]. This was later presented and implemented by the Swedish Institute for Infectious Disease Control (*Smittskyddsinstitutet*, henceforth SMI) in 2013, and the National Board of Health and Welfare quickly followed in how they announced that persons living with HIV could now get their ‘duty to inform’ removed, if they were under effective treatment and did not live with any other sexually transmitted diseases [31, 114]. The Swedish Institute for Infectious Disease Control later merged with the Public Health Agency of Sweden (FoHM) in 2014. As SMI confirmed in their investigation from back then, the risk of transmission is minimal if the person in question is under effective ART. This estimation concerns anal, oral, and vaginal intercourse, and it applies regardless of gender, sexual identity, or type of sexual contact [114]. If ART is to be considered effective and stabilised, it must follow certain criteria. Firstly, the virus level inside the blood plasma must continuously be lower than 50 copies per ml (as the virus level is considered immeasurable if it falls under 50 copies), which must have been verified at two consecutive occasions within a three to six months interval [114]. Also, the patient in question must be deemed to have a continuously high treatment-compliance. As described in the previous quote from the interview with RFSU, the interpretation of the law in Sweden changed in 2013–2014, much in response to these findings. The treating physician is allowed to exempt the HIV-positive person from the duty-to-inform (about one’s HIV status) in sexual contacts, provided that they 1) have an undetectable plasma viral load; 2) are therapy compliant; and 3) are not known to have another sexually transmitted disease [110, 111, 115, 116]. Whereas i.e., the US Centers for Disease Prevention and Control uses the cut-off of <200 copies for viral load to be considered “undetectable”, since 2018, Swedish law utilises the cut-off of <50 copies after an HIV-positive man was found not guilty of danger to others in the Supreme Court of Sweden. The man in question had had unprotected sex with his partner, but was found not guilty due to his undetectable plasma viral load and therapy compliance [38]. In sum, there is a noticeable loosening in the approach towards the duty-to-inform [Table 3]. Albeit not so much, because it appears counterproductive; it being considered less necessary due to current medical developments.

**Table 3:** Flow chart of the various key changes and dates on the mandatory HIV reporting.

1981	First reported case of HIV diagnosis.
1982	First (official) diagnosed case of HIV in Sweden (Roar Klingenberg).
1987	The Act on Banning Gay Sauna Clubs (SFS 1987:375) enacted in Sweden.
1988	The Communicable Diseases Act revised (from SFS 1968:231) to incorporate HIV/AIDS, including a mandatory partner notification (SFS 1988:1472).
2004^a^	^a^ The Act on Banning Gay Sauna Clubs lifted.
2004^b^	^b^ The Communicable Diseases Act is revised yet again (SFS 2004:168), to accommodate the lifted ban on gay sauna clubs; yet mandatory partner notification remains the same.
2006	The Swedish National Strategy on HIV/AIDS is officially implemented.
2008	The Swiss National Commission on AIDS presents new data that shows how individuals with undetectable viral load (and no STI) cannot transmit HIV during sex.
2013	Swedish authorities publish report that reaffirms data from the Swiss National Commission on AIDS.
2017	The Swedish National Strategy on HIV/AIDS is revised.
2018^a^	^a^ The Supreme Court of Sweden sets new precedent; an HIV-positive man is found not guilty (of danger to others in having unprotected sex) due to his undetectable plasma viral load and therapy compliance.
2018^b^	^b^ Physicians in Sweden may now remove HIV-positive patients’ mandatory partner notification—if they have an undetectable plasma viral load and are therapy compliant.
2019	First motion to propose a permanent removal of the mandatory partner notification is presented to the Swedish Parliament.
2022	The Swedish Parliament proposes another revision of the Swedish National Strategy on HIV/AIDS.

## Discussion

### The importance of stigma reduction

HIV/AIDS has been a persistent global health challenge, but advancements in medical knowledge and treatments have helped to reduce the stigma associated with the virus. However, despite progress, stigma continues to impact the lives of individuals living with HIV. Apropos the historical trajectory, this article also seeks to address the role of stigma in HIV prevention and treatment, highlighting its effects on testing, treatment adherence, and the use of pre-exposure prophylaxis (PrEP). Stigma surrounding PrEP users, particularly within the gay community, can discourage individuals from using this effective preventive measure. Furthermore, disclosure of HIV status remains a concern due to fears of rejection or mistreatment. The detrimental effects of stigma on the mental, sexual, and physical well-being of people living with HIV are evident [117–119], highlighting the significance of diminishing stigma as a dual approach—limiting the virus’s transmission and enhancing the well-being of those affected by it [119–121]. Despite the limited research on interventions aimed at diminishing HIV stigma in both Sweden and worldwide, it is imperative to tackle stigma across different tiers, encompassing peer support networks, training for healthcare providers, and policy reforms. This comprehensive approach is vital for attaining improved health results and fostering inclusiveness for individuals living with HIV.

Intrinsically, it may be that a further step in preventing the spread of HIV could be taken through stigma reduction. As this article demonstrates, stigma can discourage people from getting tested and receiving timely treatment. Moreover, acquiring HIV can be prevented by as much as 99 percent when a person takes a PrEP pill (pre-exposure prophylaxis) every day [111]—but again, stigma can easily emerge. Namely, PrEP users are commonly stereotyped as sexually irresponsible, promiscuous, and immoral individuals [122] who use the drugs as a ‘license’ or free pass to have casual unprotected sex. For example, the term “Truvada Whore” (after *Truvada*, a brand of emtricitabine/tenofovir) quickly went viral and was widely used in the United States to refer to a man who has sex with other men and uses PrEP as internal HIV prevention. Other equally effective PrEP medications available are Descovy (TAF/FTC) and cabotegravir injections (Apretude). However, while the term came to be as a way of shaming gay men, it was later appropriated by the gay movement and used as a way of expressing agency [123, 124]. That said, the term has now grown rather obsolete and is rarely heard or used anymore. Yet nonetheless, such stigmas can lead to people being less willing to using PrEP in the first place, or less faithful in continuously taking it [111, 122, 125].

Although the new medical advancements and knowledge about HIV/AIDS seem to have led to less stigma than previously experienced [126, 127], the stigmas that permeate the virus have not been completely eliminated. More recent studies from Sweden [115, 116, 128, 129] suggest that persons living with HIV are still selective in their disclosures for fear of being defined by others primarily by one’s HIV status, or of being rejected or abandoned by sexual partners, or even badly treated by health care staff [117, 118, 130]. In fact, as Carlsson-Lalloo and colleagues document in their study [115], people with HIV themselves struggled to believe that with the virus level being low, so is the risk for transmission.

Tobias Karlsson, a Swedish professional dancer who publicly disclosed his HIV status in 2019, expressed something similar in an interview with LGBTQIA+ magazine *QX*, all while expressing his surprise for how widespread the ignorance is apropos how medical advancements have actually transformed the illness experience:

I had no idea that [when the virus level is so low] there is no risk for transmission during sex … When one says that one is ‘undetectable’; when the virus level is immeasurable, people are fine with it. You are no longer considered ill and cannot pass on the virus, where having sex is considered safe, i.e., being HIV-positive under medication and with an undetectable virus level. And you no longer have to disclose your HIV status to a potential sexual partner. [131]

Yet hearing that one’s viral load is suppressed and that the risk of transmission is even ‘effectively zero’ (as *Undetectable = Untransmittable*), simultaneously as one attains a deeper understanding of the individual infectivity rate and risk of transmission, “seems to reduce feelings of being contagious” [115]—thereby also effectively preventing experiences of self-stigma. However, within the gay community itself, stigma is largely experienced and affects people not only when they personally face negative judgments—but also when they hear or read negative thoughts and opinions about other persons living with HIV [128, 129, 132]. Outside the gay community, gay men living with HIV are faced with the intersectional stigma of being a sexual minority, as well as having HIV.

Stigma negatively affects the mental, sexual, and physical well-being of persons living with HIV. Improving their health-related quality of life is, therefore, cited as “the fourth 90” that should be achieved [132] within the Joint UNAIDS/WHO continuum of HIV care targets. Hence, reducing stigma would serve the dual purpose of reducing the spread of HIV and improving the quality of life of persons living with the virus. However, no research has yet been done on interventions to reduce HIV stigma in Sweden; and international research within this area is also very limited [11, 12, 107]. Needless to say, there is still a great deal of improvement to be achieved in this regard. The available research suggests, among other things, that reducing stigma can be done at different levels—micro, mezzo, and macro. For example, at micro level self-stigma can be countered through various peer-support systems; at health care level (macro), interventions can target health-care providers explicitly [107, 130, 132]. At policy level (mezzo), enacting decriminalised HIV legislation is not only recommended—but also directly necessary, if the good levels of quality of life and self-rated health for persons with HIV are to improve at all.

This article delves into a myriad of crucial dimensions pertaining to HIV disclosure, sexual well-being, and the broader societal context. Notably, it casts a spotlight on Sweden; a nation that achieved the pioneering Joint UNAIDS/WHO 90-90-90 continuum. On a global canvas, exemplified by i.e., the United States, the presence of legislation mandating the divulgence of one’s HIV-positive status to both sexual and drug-sharing partners underscores a resolute commitment to curbing viral dissemination and safeguarding the health of those potentially vulnerable [23]. This legal framework underscores a shared responsibility to curtail transmission risks. In parallel, Sweden’s embrace of the U = U (*Undetectable = Untransmittable*) mantra marks a progressive stride forward. This stance acknowledges the scientifically established premise that an individual boasting an undetectable viral load due to effective HIV treatment poses no discernible risk of transmitting the virus through sexual contact. Consequently, for those bearing this status, the onus to disclose is mitigated, reflecting a harmonisation of policy with medical wisdom.

In this intricate landscape, the Joint United Nations Programme on HIV and AIDS (UNAIDS) and the World Health Organization’s (WHO) guidelines on “beneficial disclosure” [133] prominently underscore the ethical mandate for well-informed and mutually agreed-upon choices in the realm of sexual health. Affirming the rights and well-being of HIV-negative partners, these guidelines accentuate the paramountcy of transparency and candid dialogue within intimate relationships. Nevertheless, the article raises a pertinent ethical quandary through an illustrative scenario. It probes whether withholding one’s status—a non-disclosure—could indeed be hailed as a “tremendous victory”, as intermittently articulated in the article, for a potentially affected partner. This underlines the pivotal significance of informed consent and individual agency in the realm of intimate relationships, where every individual deserves the autonomy to shape decisions grounded in accurate information.

Accentuating the weight of open conversations between sexual partners concerning protective measures such as condoms and pre-exposure prophylaxis (PrEP), it is imperative to advocate for sincere discussions that duly honour the autonomy and preferences of all stakeholders. Such a stance acknowledges the synergy between responsible sexual conduct and forthright communication regarding HIV status. Nevertheless, the article undertakes a broader exploration of the repercussions of HIV criminalisation, notably spotlighting Sweden’s context. Such criminalisation potentially dissuades individuals from seeking crucial HIV-related services. The equilibrium between this concern and the imperative to avert transmission constitutes a nuanced challenge, necessitating a meticulous and comprehensive approach bridging legal and public health realms. In the final analysis, the article ardently underscores the need to highlight the significance of voluntary HIV status disclosure, aligning harmoniously with the bedrock principles of ethical decision-making and reverence for individual autonomy. Within the discourse encompassing HIV status revelation, the centrality of the well-being and rights of all stakeholders should be unwavering, ensuring that enlightened choices materialise within an ambiance of unreserved communication and shared understanding.

### Beneficial disclosure and partner notification

The guidelines set forth by UNAIDS and WHO [133] pertaining to beneficial disclosure and ethical partner counselling hold considerable relevance for their application in the context of Sweden’s HIV/AIDS policies. By aligning with these guidelines, Sweden can further enhance its efforts to address the complexities of HIV/AIDS, promote individual autonomy and dignity, and bolster public health outcomes. The principles of voluntary, respectful, and confidential disclosure articulated by UNAIDS and WHO underscore the importance of providing individuals with the agency to decide when and how to disclose their HIV status. This aligns with Sweden’s commitment to individual rights and personal privacy [37]. Enabling individuals to make informed decisions about sharing their HIV status not only respects their autonomy but also contributes to the creation of a supportive environment that can lead to more open discussions about HIV/AIDS within the community. The emphasis on beneficial outcomes for all parties involved—the individual, their sexual and drug-injecting partners, family, and the broader community—resonates with Sweden’s inclusive approach to healthcare [37]. By encouraging disclosure that results in better health and informed decision-making for both uninfected and infected individuals, Sweden can foster a culture of responsibility and collaboration.

When considering partner counselling (partner notification), the ethical principles outlined by UNAIDS and WHO [133] can guide Sweden’s policies in a balanced manner. Prioritising informed consent and confidentiality while recognising the potential consequences of not notifying partners aligns with Sweden’s commitment to ethical healthcare practices [37]. The emphasis on professional efforts to persuade and support HIV-positive individuals in notifying their partners mirrors Sweden’s emphasis on comprehensive and patient-centred care. In cases where an HIV-positive person refuses to counsel their partners despite thorough efforts to persuade them, Sweden can draw insights from the guidelines’ approach to this complex situation. Ethical weighing of potential harms and informed decision-making can ensure that partner counselling is pursued responsibly. The emphasis on protecting individuals from abuse, discrimination, and stigma resonates with Sweden’s strong stance on human rights and equality [37]. Adapting the UNAIDS and WHO guidelines [133] in Sweden would necessitate clear policy frameworks, legal protections, and comprehensive training for healthcare workers and counsellors. By enacting policies that safeguard confidentiality and informed consent, Sweden can establish a supportive environment for ethical partner counselling. Additionally, training initiatives would equip healthcare professionals with the skills and sensitivities required to engage in these delicate discussions effectively.

Overall, embracing the UNAIDS and WHO guidelines on beneficial disclosure and ethical partner counselling can elevate Sweden’s efforts in HIV/AIDS prevention and care. These principles align closely with Sweden’s commitment to human rights, individual autonomy, and public health. By integrating these guidelines into its healthcare system, Sweden can reinforce its position as a leader in progressive and compassionate HIV/AIDS policies.

## Conclusion

The emergence and transmission of the HIV viruses can be attributed to various factors, including population movement, urbanisation, changes in sexual relations, new medical procedures, and even war [4]. This signifies that the AIDS pandemic is not solely of zoonotic origin (SIV; simian immunodeficiency virus) but rather a disaster influenced by complex human factors beyond mere sexual behaviour, such as political decisions and anti-science sentiments. As such, it remains significant to highlight the importance of considering various factors when discussing public health challenges. Despite Sweden not experiencing a severe epidemic and being the first country to achieve the 90-90-90 continuum of care targets, HIV and AIDS have still resulted in significant stigma. This stigma has been perpetuated by publicised incidents, strong opinions, and policies that criminalise gay men living with HIV, ultimately viewing them as a risk rather than recognising them as victims.

The impact of punitive and criminalising legislation and attitudes has proven to be counterproductive in preventing the spread of HIV. Instead, such legislation fosters stigma and fear, hindering preventive efforts, early detection, and treatment. Fortunately, there have been positive developments in recent years. Medical advancements have contributed to a shift in attitudes, exemplified by the Swedish Parliament’s decision in 2019 to investigate a potential abolishment of the obligation to disclose one’s HIV status [134, 135]. In response, the Swedish government tasked the Public Health Agency of Sweden with looking into updating the Swedish National HIV/AIDS Strategy (with certain respect to mandatory partner notification), with results expected by April 2023 [136]. This move represents an important milestone, as it not only improves the quality of life for gay men living with HIV but also eliminates the risk of being viewed as criminals solely based on their HIV status.

In addition to legislative changes, it is crucial to implement anti-stigma interventions that aim to enhance the well-being of gay men living with HIV and further improve HIV prevention in Sweden. By challenging societal prejudices and fostering understanding, these interventions can contribute to a more supportive and inclusive environment. Ultimately, the abolition of the obligation to disclose HIV status stands as a significant victory for all individuals affected by HIV, eliminating the stigma and empowering those living with the virus to lead fulfilling lives without fear of criminalisation. After all, there is limited evidence to support the effectiveness of criminalisation and penal laws in reducing HIV incidence. On the contrary, such laws may undermine the prevention efforts outlined not only in Sweden’s National HIV/AIDS Strategy but also in national strategies worldwide.
